# Sudden-onset diffuse cutaneous hyperpigmentation as an unusual early sign of systemic sclerosis

**DOI:** 10.1016/j.jdcr.2025.08.019

**Published:** 2025-08-29

**Authors:** Raqiya AL. Rajaibi, Aya Al lamki, Rihab Al Abri, Humaid Al Wahshi, Mohammed Al Masqari

**Affiliations:** a Dermatology Department, Rustaq Polyclinic, Rustaq, Oman; b Oman Medical Specialty Board, Muscat, Oman; c Dermatology Department, Medical City For Military and Security Service, Muscat, Oman; d Department of Rheumatology, Royal Hospital, Muscat, Oman; e Department of Histopathology, Royal Hospital, Muscat, Oman

**Keywords:** systemic sclerosis, pigmentation, hyperpigmentation, hypertension, case report, Oman

## Introduction

Systemic sclerosis (SSc), also known as scleroderma, represents a rare group of fibrosing autoimmune connective tissue disorders with variable organ involvement and cutaneous manifestations. Although skin induration—the hardening or thickening of the skin due to increased fibrous tissue—is the main cutaneous symptom, it can be absent in some patients, making the diagnosis more challenging.^1^ Pigmentary abnormalities have been described, but sudden-onset, diffuse hyperpigmentation without skin sclerosis is an unusual and misleading early indicator.^1^ Although SSc cannot be cured, therapies are available to alleviate symptoms, decelerate disease advancement, and enhance overall well being.^2^ This case report describes a 58-year-old woman with SSc who presented with hypertension and sudden-onset, progressive, diffuse cutaneous hyperpigmentation.

## Case report

**Fig 1:**
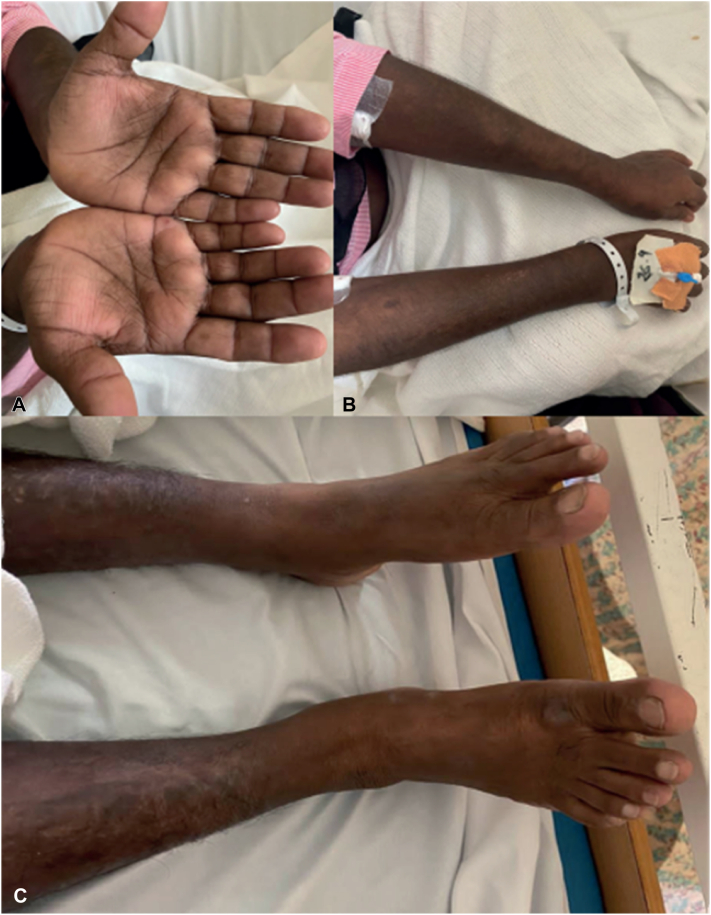
Clinical photographs of a 58-year-old woman with diffuse cutaneous hyperpigmentation over the **(A)** palms and **(B)** upper and **(C)** lower limbs.

**Fig 2:**
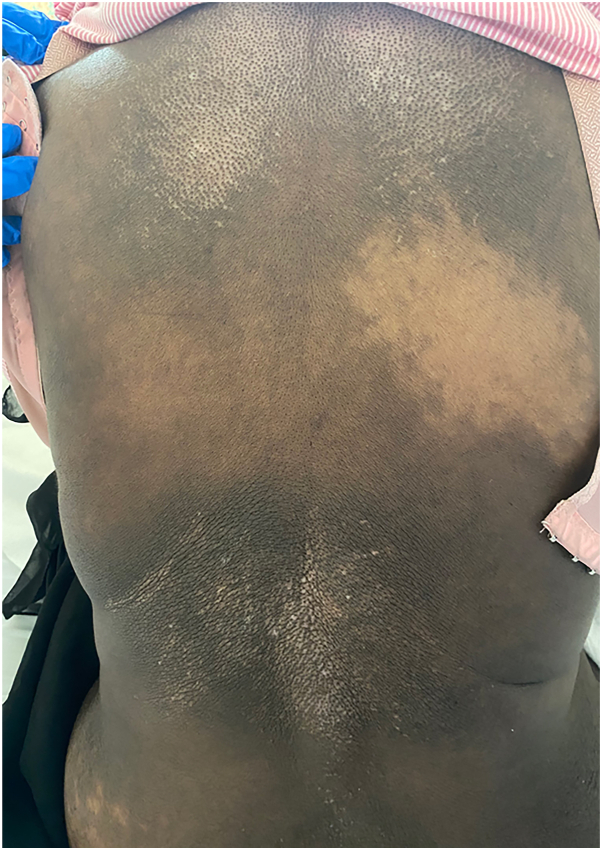
Clinical photograph of salt-and-pepper sign over the patient's back.

A 58-year-old woman with no prior relevant medical history was admitted to the hospital due to hypertension. She presented with sudden-onset, progressive, diffuse cutaneous hyperpigmentation, which initially appeared over her trunk and abdomen before spreading to her limbs and face over the course of 1 month. Aside from occasional epigastric discomfort, a review of systems was unremarkable. Upon physical examination, the patient was found to have markedly elevated blood pressure (∼182/104 mmHg) and diffuse hyperpigmentation across her face, trunk, and limbs (Fig 1). Additionally, an area of hypopigmentation with perifollicular darkening, resembling a salt-and-pepper pattern (Fig 2), was observed over her back. However, there was no evidence of skin thickening or any changes to the nails or mucous membranes.

Laboratory tests revealed an anti–nuclear antibody (ANA) reactive titre of 1:320 with a speckled staining pattern. A full workup was within normal ranges, including a complete blood count, renal, liver, and thyroid function tests, as well as cortisol and ferritin levels. Results were negative for a panel of extractable nuclear antigen antibodies, including anti–centromere, anti–Scl70, and anti-ribonucleoprotein (anti-RNP) antibody, as well as negative for anti–neutrophil cytoplasmic antibodies. The anti–RNA polymerase III antibody test was not available. Urinalysis revealed proteinuria and a high protein-to-creatinine ratio. A chest x-ray showed cardiomegaly, whereas an echocardiogram revealed mild pulmonary hypertension and minimal pericardial effusion. Pan–computed tomography indicated a dilated esophagus with diffuse thickening at the gastro-esophageal junction and lungs, with areas of interlobular septal thickening and subpleural ground glass opacity.

**Fig 3:**
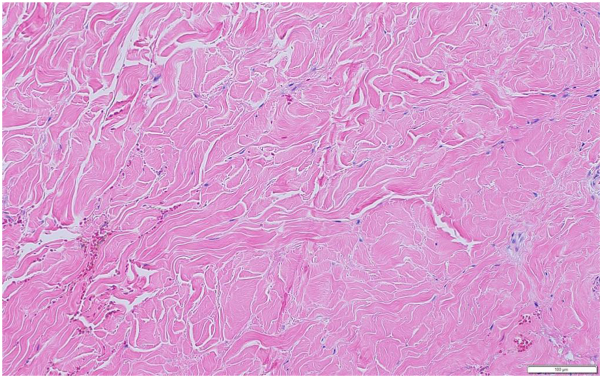
Skin biopsy showing the widespread presence of thick collagen bundles in the dermis, along with the replacement of fat around the sweat glands with collagen. Mild inflammation is also evident (Haematoxylin and eosin stain; original magnification: ×40).

Following the initial assessment, the patient was scheduled for an oesophagogastroduodenoscopy, spirometry, and high-resolution computed tomography imaging. Histologic analysis of a skin punch biopsy taken from the left forearm revealed notable thickening of collagen bundles in the dermis, with collagen replacing adipose tissue around the sweat glands, as well as mild inflammation (Fig 3). Subsequently, the patient was referred to the nephrology team for a renal biopsy, which demonstrated focal segmental glomerulosclerosis and moderate arteriosclerosis. Both the skin and renal biopsy findings were consistent with a diagnosis of SSc with renal involvement. A follow-up consultation with the rheumatology team was advised for further assessment and investigation to rule out paraneoplastic syndromes. Unfortunately, the patient failed to attend subsequent appointments due to COVID-19 pandemic and died a few months later due to pulmonary hypertension-associated SSc.

## Discussion

Overall, SSc is a rare autoimmune disease of unknown etiology that affects multiple organs. It is characterized by skin and organ fibrosis, vasculopathy, and immune activation involving autoantibodies such as anticentromere, anti–topoisomerase I, and anti–RNA polymerase III.^3^ The concept of early SSc, first introduced by Koenig et al^4^ in 2008 and later validated by the American College of Rheumatology and the European League Against Rheumatism in 2013, has facilitated our understanding of disease progression.^1^^,^^4^ Typically, SSc is classified into diffuse and limited subtypes based on the extent of skin involvement. Diffuse SSc is marked by extensive skin thickening, although limited SSc is confined to the distal extremities. These subtypes exhibit distinct clinical courses, rates of progression, and patterns of organ involvement.^1^^,^^3^

Cutaneous manifestations of SSc tend to occur more frequently and precede manifestations in other organs. Key cutaneous signs include Raynaud’s phenomenon—a condition in which blood vessels in the fingers and toes temporarily narrow in response to cold or stress, causing a change in skin color and sensation—as well as dyspigmentation, digital ulcers, cutaneous sclerosis, telangiectasias, calcinosis cutis, and pruritus.^4^^,^^5^ However, the absence of cutaneous sclerosis can complicate diagnosis, necessitating thorough investigations for confirmation. In cases in which scleroderma cutaneous manifestations are absent, SSc is termed systemic sclerosis sine scleroderma. This designation includes patients with Raynaud’s phenomenon, positive ANA results, and involvement of at least 1 visceral organ typical of SSc. The first documented case of visceral SSc without evident skin involvement was reported by Abrams et al^6^ in 1954.

Dyspigmentation, often an early indicator of SSc, can aid in diagnosis and potentially indicate disease severity.^7^ Various dyspigmentation patterns in SSc have been documented in the literature, including diffuse generalized and photo-accentuated hyperpigmentation, localized depigmentation resembling vitiligo with perifollicular hyperpigmentation, or the characteristic “salt-and-pepper” appearance indicating skin sclerosis.^8^^,^^9^ Other reported patterns include streaky hyperpigmentation over cutaneous blood vessels in areas of localized hypopigmentation as well as reticulate hyperpigmentation, characterized by a net- or lace-like pattern on the skin.^10^, ^11^, ^12^ Despite affecting approximately 50% of patients, the precise pathogenesis of hyperpigmentation in SSc remains unclear.^10^ Some theories propose that fibroblasts, the primary cell type implicated in fibrosing disorders, may influence epidermal pigmentation during extracellular matrix production.^13^ This process is mediated by fibroblast growth factors, along with other growth factors such as melanocyte-activating factors, such as stem-cell factors, and endothelin.^14^^,^^15^

Early SSc typically presents with Raynaud’s phenomenon, SSc-related autoantibodies, and/or capillaroscopic abnormalities, none of which were evident in the current patient.^4^ However, despite not meeting all of the aforementioned criteria, the patient described in this study exhibited skin hyperpigmentation, positive ANA results, skin biopsy findings, and evidence of multisystemic involvement, including renal, cardiopulmonary, and gastrointestinal systems, thereby leading to the final diagnosis of SSc.

## Conclusion

Diffuse hyperpigmentation encompasses a broad differential diagnosis, necessitating a targeted diagnostic approach to refine potential aetiologies. Cutaneous manifestations are frequently the earliest indicators of SSc, underscoring the dermatologist’s pivotal role in ensuring accurate diagnosis and coordinating multidisciplinary management. Early identification of SSc is imperative for optimal care due to the risk of potentially fatal involvement of visceral organs, such as the heart, lungs, kidneys, and gastrointestinal tract.

## Conflicts of interest

None disclosed.
